# Shortening of Zika virus CD-loop reduces neurovirulence while preserving antigenicity

**DOI:** 10.1371/journal.pntd.0007212

**Published:** 2019-03-07

**Authors:** Kenneth H. Dinnon III, Emily N. Gallichotte, Ethan J. Fritch, Vineet D. Menachery, Ralph S. Baric

**Affiliations:** 1 Department of Microbiology and Immunology, University of North Carolina at Chapel Hill, Chapel Hill, North Carolina, United States of America; 2 Department of Microbiology and Immunology, University of Texas Medical Branch, Galveston, Texas, United States of America; 3 Department of Epidemiology, University of North Carolina at Chapel Hill, Chapel Hill, North Carolina, United States of America; Fundaçao Oswaldo Cruz, BRAZIL

## Abstract

Zika virus (ZIKV) is a mosquito-borne positive sense RNA virus. Recently, ZIKV emerged into the Western hemisphere as a human health threat, with severe disease associated with developmental and neurological complications. The structural envelope protein of ZIKV and other neurotropic flaviviruses contains an extended CD-loop relative to non-neurotropic flaviviruses, and has been shown to augment ZIKV stability and pathogenesis. Here we show that shortening the CD-loop in ZIKV attenuates the virus in mice, by reducing the ability to invade and replicate in the central nervous system. The CD-loop mutation was genetically stable following infection in mice, though secondary site mutations arise adjacent to the CD-loop. Importantly, while shortening of the CD-loop attenuates the virus, the CD-loop mutant maintains antigenicity in immunocompetent mice, eliciting an antibody response that similarly neutralizes both the mutant and wildtype ZIKV. These findings suggest that the extended CD-loop in ZIKV is a determinant of neurotropism and may be a target in live-attenuated vaccine design, for not only ZIKV, but for other neurotropic flaviviruses.

## Author summary

Zika virus (ZIKV) is a mosquito-transmitted virus that was recently introduced in Brazil and subsequently spread throughout the Americas. ZIKV is highly similar to the related dengue virus but causes unique disease outcomes including neurological disease in adults and fetal developmental complications. The ZIKV envelope protein coats the surface of the virus and allows entry into host cells. Here we investigate a portion of the ZIKV envelope protein, the CD-loop, which extends further than in dengue virus, and its role in ZIKV neurological disease. Our study finds that shortening the CD-loop reduces the ability of ZIKV to replicate in neuronal cells, that the longer CD-loop is a key factor for invasion of the central nervous system in mice, and that a deletion in the CD-loop is genetically stable with passage. Additionally, we show that infection with the CD-loop mutant induces a potent antibody response that can neutralize wildtype ZIKV, suggesting it may offer protection in mice. Shortening of the ZIKV CD-loop, and the CD-loop of other neurotropic flaviviruses, could contribute to development of rapid, effective, and safe vaccines.

## Introduction

Zika virus (ZIKV) is a positive-sense single-stranded RNA flavivirus. Though first isolated in 1947 and after years of relatively benign infections throughout Southeast Asia, ZIKV was identified in large human disease outbreaks in Yap Island, French Polynesia, and then the Americas [1–5]. ZIKV infection is asymptomatic in a majority of adult cases, but when symptomatic, generally causes mild febrile illness [6, 7]. ZIKV infection has also been associated with more severe disease, such as neurological complications including Guillain-Barré syndrome [7–10]. Additionally, ZIKV infection during pregnancy is linked to microcephaly and fetal demise [11, 12].

The ability of ZIKV to cause neurological disease is not unique among flaviviruses [13]. West Nile virus (WNV), Japanese encephalitis virus (JEV), and tick-borne encephalitis virus (TBEV) also infect the central nervous system (CNS) and cause encephalitic disease. However, dengue virus (DENV), a highly similar virus both genetically and antigenically, only very rarely causes neurological or encephalitic disease [14, 15], highlighting a disconnect between disease and virus relatedness. While the complete viral determinants of flavivirus neurotropism are not fully known, there are several described conserved and divergent mechanisms [13]. For example, multiple groups have shown that envelope protein glycosylation is necessary for neurovirulence of WNV and ZIKV [16–19]. However, this glycosylation motif is conserved between both neurotropic and non-neurotropic flaviviruses, indicating that it is not solely sufficient for neurotropism. Additionally, several groups have identified novel neuronal ZIKV receptors, such as AXL, that are not utilized by other neurotropic flaviviruses [20, 21].

Multiple groups have shown that ZIKV is more structurally stable than DENV, hypothesizing that it can persist longer in body compartments and fluids, potentially leading to an increased chance of neuroinvasion [22–25]. One key difference between the structural envelope proteins of neurotropic and hemorrhagic flaviviruses is the extension of the CD-loop by a single amino acid [23, 24]. Though predicted to stabilize the virus via a network of hydrogen bonds, we have previously shown that the extended loop itself, independent of hydrogen bonding, is responsible for ZIKV’s structural and thermal stability [23, 24]. Additionally, shortening of the CD-loop by a single amino acid (Δ346 ZIKV) attenuated the virus in a mouse model of ZIKV pathogenesis [24]. Interestingly, while both viruses replicate in the periphery, the CD-loop mutant was less likely to be found in the CNS of infected mice compared to wildtype (WT) ZIKV. This suggests that the extended CD-loop, or the structural stability that it confers, is important for neuropathogenesis.

In this study we further investigate the role of the ZIKV CD-loop in neurotropism and antigenicity. We find that the CD-loop mutant is delayed in disseminating to the brain, but once present can replicate and cause lethal disease. Importantly, when delivered via intracranial infection, Δ346 ZIKV is still less pathogenic, indicating that the attenuated phenotype is not solely due to a defect in neuroinvasion. While the Δ346 deletion is genetically stable after long-term *in vivo* infection, secondary site mutations emerge in the envelope glycoprotein. A successful ZIKV vaccine should have no risk of neurological complications in immunocompetent populations, and as our shortened CD-loop virus results in decreased neuropathogenicity, we characterized the antibody response elicited by the Δ346 virus in mice. Mice infected with the Δ346 mutant mount an antibody response that similarly neutralizes both Δ346 and WT ZIKV in cell culture. Together, this data suggests that shortening the CD-loop of ZIKV results in decreased neurovirulence while maintaining wildtype ZIKV antigenicity.

## Results

### ZIKV CD-loop mutant is attenuated in a neuronal cell line

**Fig 1:**
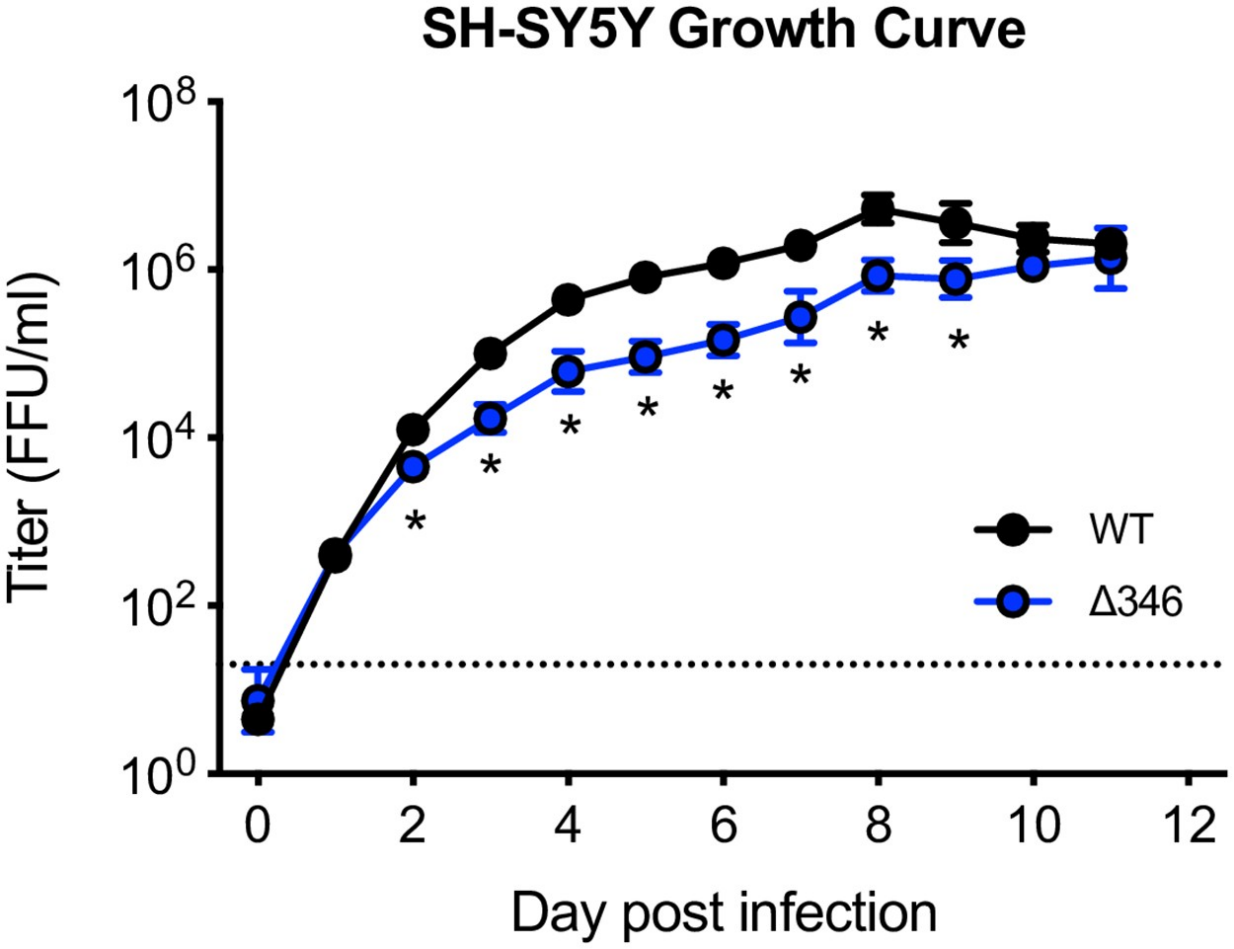
Δ346 mutant is attenuated in neuronal cell line. Growth curve of SH-SY5Y cells infected at an MOI of 1 in triplicate wells. Infectious virus titer (FFU/ml) was determined on C6/36 cells. Two-factor ANOVA followed by Sidak’s multiple comparison was conducted on log-transformed data. * represents *p* < 0.05. Dotted line represents limit of detection. Data points not detected are plotted at half the limit of detection. Error bars represent standard deviation.

We have previously shown that shortening of the ZIKV CD-loop results in attenuated replication in both mammalian Vero and mosquito C6/36 cells [24]. To determine if the Δ346 virus is attenuated in neuronal cells, we performed a single step growth curve in the human neuroblastoma cell line SH-SY5Y, which have been previously used as an *in vitro* neurological model of ZIKV replication (Fig 1) [26–28]. Though Δ346 ZIKV replicated similarly to WT ZIKV at early time points, the Δ346 virus was slightly attenuated throughout most of the time course before finally reaching equivalent peak titers (p<0.05). This indicates that despite minor attenuation relative to WT ZIKV, the CD-loop mutant is capable of infection and replication in neuronal cells.

### Δ346 virus can reach and replicate in the brain in immunocompromised mice

**Fig 2:**
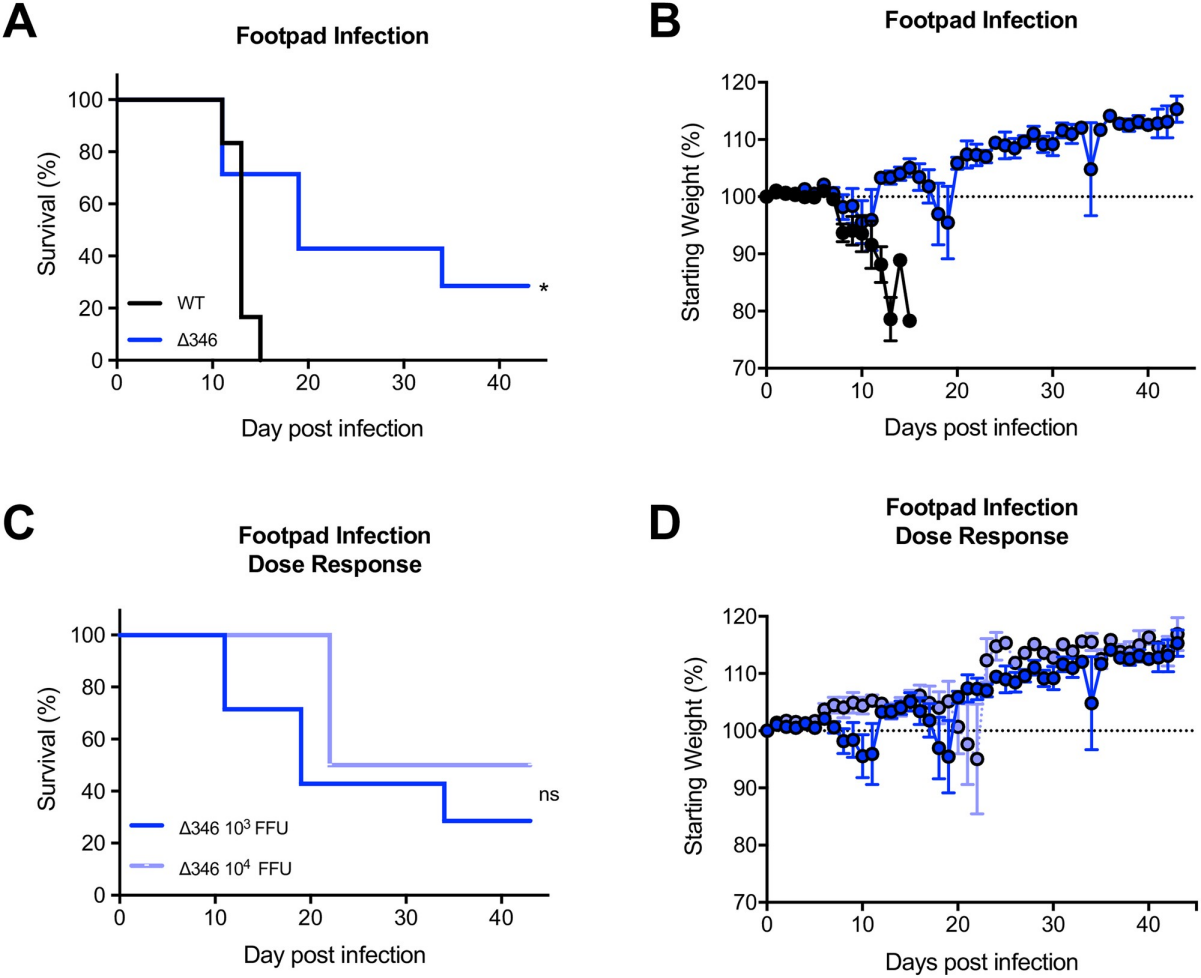
Δ346 virus is attenuated in IFNAGR^-/-^ mice. (A-B) IFNAGR^-/-^ mice were infected with 10^3^ FFU of WT or Δ346 ZIKV via footpad infection and monitored for (A) survival and (B) weight loss (n = 13 for each group). (C-D) Mice were infected with 10^3^ or 10^4^ FFU Δ346 ZIKV via footpad infection and monitored for (C) survival and (D) weight loss (n = 13 and 4, respectively). Log-rank test was conducted on survival curves. * represents *p* < 0.05. ‘ns’ represents not statistically significant. Dotted line represents 100% starting weight. Error bars represent standard error of the mean.

**Fig 3:**
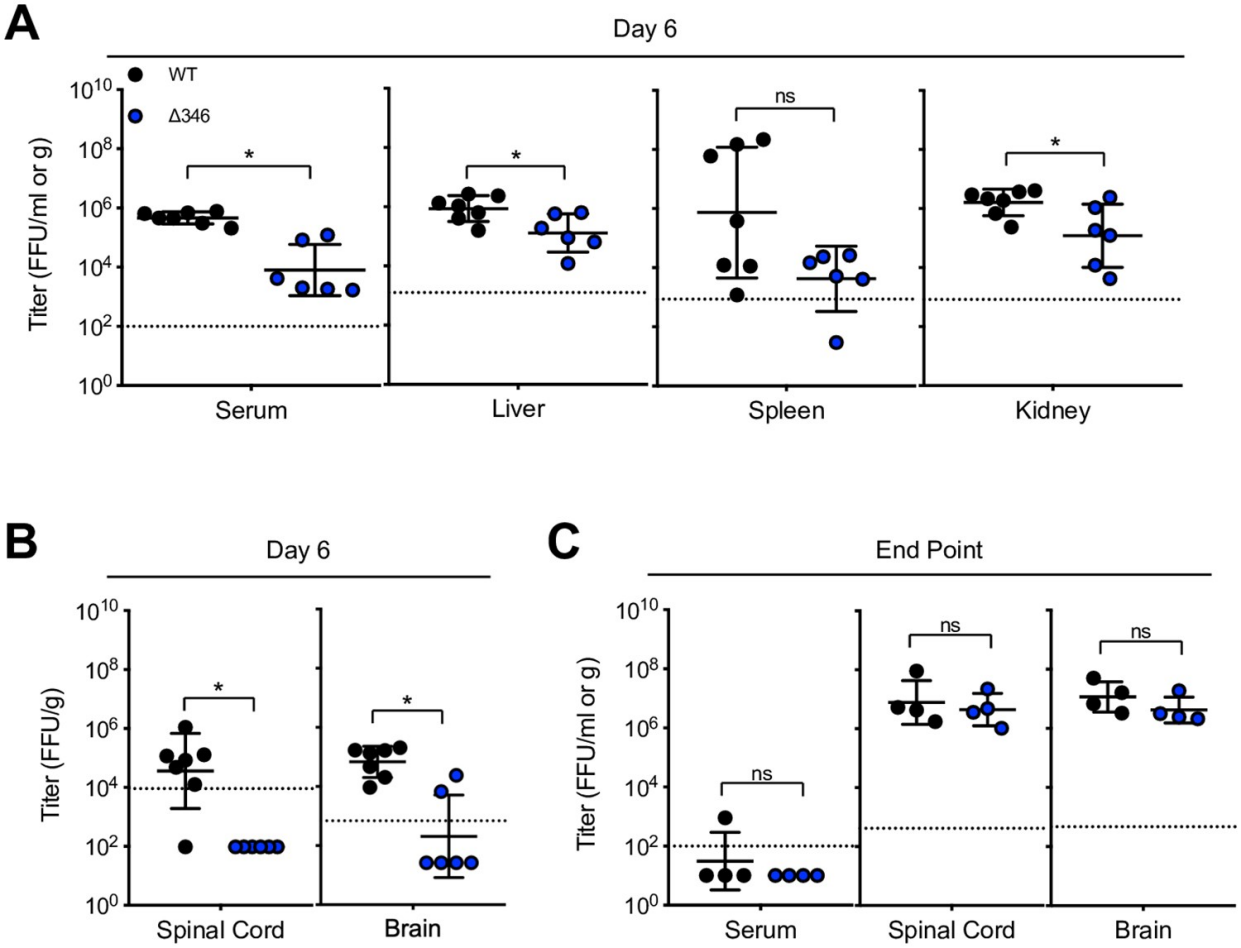
Δ346 mutant is attenuated early but reaches similar end point titers in immunocompromised mice. IFNAGR^-/-^ mice were infected with 10^3^ FFU of WT or Δ346 ZIKV via footpad infection. On day six post infection, (A) serum, liver, spleen, kidney, (B) spinal cord, and brain were harvested and titered for infectious virus levels (n = 7 WT and n = 6 Δ346 ZIKV infected mice). Moribund mice were harvested for (C) serum, spinal cord, and brain end-point titer analysis (n = 4 for each group). Mann-Whitney tests were performed on log-transformed data. * represents *p* < 0.05. Dotted line represents limit of detection. Data points not detected are plotted at half the limit of detection. Error bars represent standard deviation.

We previously explored the Δ346 mutant in 11-week-old IFNAR^-/-^ IFNGR^-/-^ mice on a C57BL/6J background (herein referred to as IFNAGR^-/-^), observing severe attenuation with no development of disease [24]. Younger mice are more susceptible to ZIKV disease [29], allowing us to study the Δ346 mutant in a more permissive model. To determine if the Δ346 ZIKV pathogenesis attenuation also occurs in younger mice, we infected 8- to 10-week-old IFNAGR^-/-^ mice and monitored weight loss, lethality and viral loads in various tissues (Figs 2–3). Mice infected with WT ZIKV all succumb to disease by day 15 post infection, whereas lethality is delayed in Δ346 ZIKV infected mice (p<0.05) (Fig 2A and 2B). Increasing the infection dose of Δ346 ZIKV from 10^3^ to 10^4^ FFU did not increase lethality or weight loss, indicating that the attenuation phenotype is not directly dose-dependent (p>0.05) (Fig 2C and 2D). On day six post infection, the Δ346 mutant replicated to lower titers in the serum, liver, spleen, and kidneys compared to WT virus (p<0.05) (Fig 3A). Additionally, at this early time point, there were fewer Δ346-infected mice with detectable virus in their brain and spinal cord compared to WT ZIKV infected mice (p < 0.05) (Fig 3B).

IFNAGR^-/-^ mice typically succumb to ZIKV infection due to neurological complications caused by viral replication in the brain [29]. Moribund infected mice were harvested for organ end point virus titers. Despite succumbing to disease at different rates (Fig 2A), nearly all WT and Δ346 ZIKV infected mice cleared serum viremia by time of death (Fig 3C). Interestingly, mice infected with either virus had comparable titers in the brain and spinal cord (p>0.05) (Fig 3C), demonstrating that although the Δ346 virus is attenuated, it can eventually replicate to high titers and cause encephalitic disease. Overall, shortening of the CD-loop results in decreased dissemination of ZIKV to the brain and delayed mortality, but it still causes neurological disease in immunocompromised mice.

### The Δ346 deletion is genetically stable in the brains of immunocompromised mice

**Fig 4:**
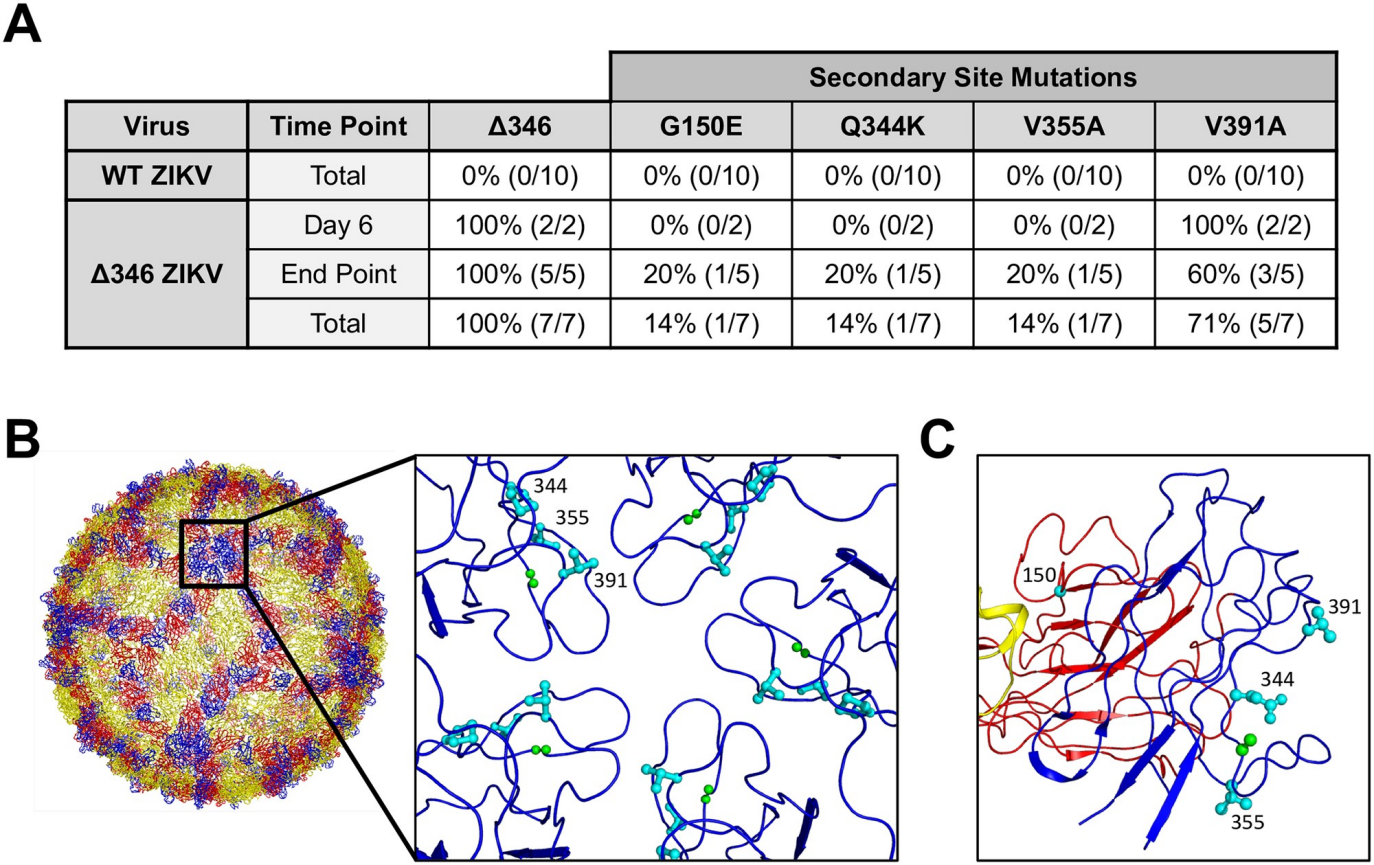
The Δ346 mutation is genetically stable in mice. The envelope gene of virus isolated from the brains of mice infected with Δ346 ZIKV via footpad was sequenced. (A) Summary table showing the status of residue 346 and secondary site mutations present at timepoints indicated. ‘*’ denotes out of mice with detectable virus in the brain. Secondary site mutations were not present in input stock virus or found in mice infected with WT ZIKV (sequences available in supplementary data file). (B-C) The location of residue 346 (green) and secondary site mutations (cyan) shown on the ZIKV envelope protein (PDB 5IZ7). (B) Top-down view of five-fold vertex or (C) side view of CD-loop on single envelope monomer.

To determine if the Δ346 virus is genetically stable in IFNAGR^-/-^ mice, we sequenced the envelope gene of virus from the brains of mice infected via footpad with WT and Δ346 ZIKV. All mice infected with the Δ346 mutant with detectible virus in their brain (Fig 3B and 3C) maintained the deletion of residue 346, demonstrating that this deletion is stable *in vivo* (Fig 4A). However, viruses in these mice had envelope protein secondary site mutations (Fig 4A) that were not detected in the virus stock (sequence available in S1 Data). At day six post infection, 100% of Δ346 ZIKV virus-positive mice contained a V391A substitution. By the end point, virus from 60% of Δ346 ZIKV infected mice contained this same substitution, indicating this residue might be important in dissemination to or replication in the CNS. Viruses from individual mice also contained three other substitutions, however each of these mutations were only present in virus from a single mouse, suggesting they are not common mutations. These secondary site mutations, with the exception of G150E, are all structurally located near the CD-loop, suggesting that they may have emerged as a consequence of the shortened CD-loop (Fig 4B and 4C). Importantly, none of these mutations emerged in mice infected with WT ZIKV (Fig 4A), revealing that these substitutions arise specifically in the context of Δ346 ZIKV. It is possible that these secondary site mutations are functionally compensating for decreased stability of the Δ346 or decreased fitness in the CNS. Future studies are necessary to elucidate the precise impact of these mutations.

### Shortened CD-loop mutant is attenuated in brain when delivered intracranially

**Fig 5:**
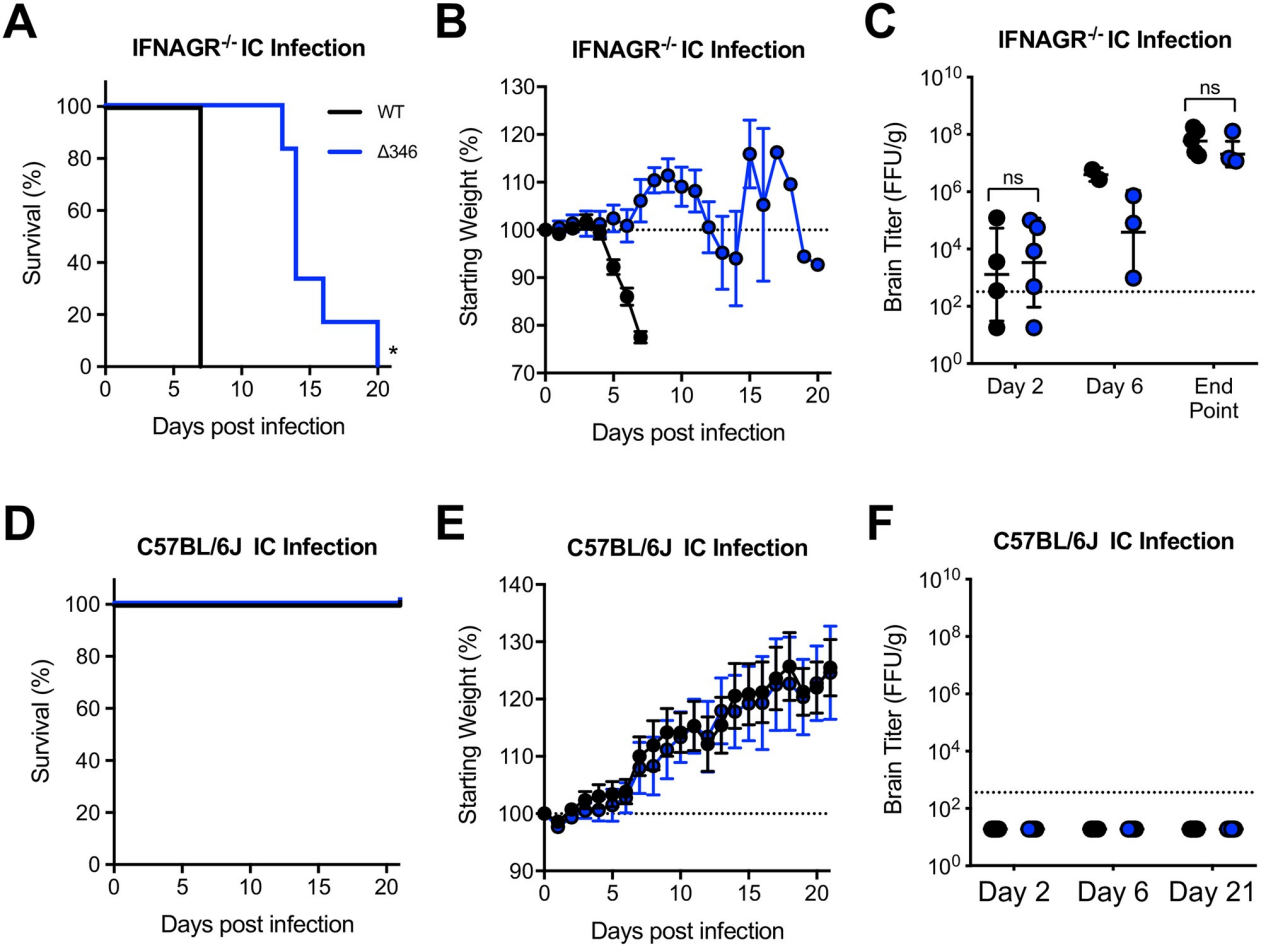
Δ346 virus is attenuated following intracranial infection. (A-C) IFNAGR^-/-^ or (D-F) C57BL/6J mice were infected with 10^3^ FFU of WT or Δ346 ZIKV via intracranial infection and monitored for (A, D) survival and (B, E) weight loss (For IFNAGR^-/-^ mice, n = 11 and 14 respectively. For C57BL/6J mice, n = 15 and 14, respectively). (C, F) On days two and six post infection, brain tissue was harvested and titered for infectious virus levels (For IFNAGR^-/-^ mice, day 2: n = 4 WT and n = 5 Δ346 ZIKV infected mice; day 6: n = 2 WT and n = 3 Δ346 ZIKV infected mice. For C57BL/6J mice, day 2: n = 4 WT and n = 5 Δ346 ZIKV infected mice; day 6: n = 4 for each group.). (C) Moribund IFNAGR^-/-^ mice and (F) on day 21 post infection, C57BL/6J mice, were harvested for end-point brain titer analysis (For IFNAGR^-/-^ mice, n = 5 for each group. For C57BL/6J mice, n = 6 for each group.). Log-rank test was conducted on survival curves. Two-factor ANOVA followed by Sidak’s multiple comparison was conducted on log-transformed titer data. * represents *p* < 0.05. Dotted line represents 100% starting weight or limit of detection. Data points not detected are plotted at half the limit of detection. Error bars represent (B, E) standard error of the mean or (C, F) standard deviation.

To determine if Δ346 ZIKV is able to replicate similarly to WT ZIKV in the brain, without the barrier of dissemination into the CNS, we performed intracranial (IC) infections in IFNAGR^-/-^ and wildtype C57BL/6J mice. Following IC injection, immunocompromised mice infected with the Δ346 mutant had delayed lethality relative to WT virus (p<0.05) (Fig 5A and 5B), but succumb to infection more rapidly than when inoculated via footpad infection (Fig 2A and 2B). CD-loop mutant infected mice have similar brain viral titers on days two and six compared, and end point to WT ZIKV infected mice (Fig 5C). Wildtype C57BL/6J mice did not show any signs of disease or viral replication in the CNS following IC infection with either virus (Fig 5D–5F). Even when delivered to the brain, an immunopriveledged site, of immunocompetent mice, neither virus is capable of causing disease. Overall, the shortened CD-loop mutant is attenuated relative to WT ZIKV at replicating the CNS, causing delayed disease in immunocompromised mice.

### Δ346 mutant induces similar antibody response as WT virus in mice

**Fig 6:**
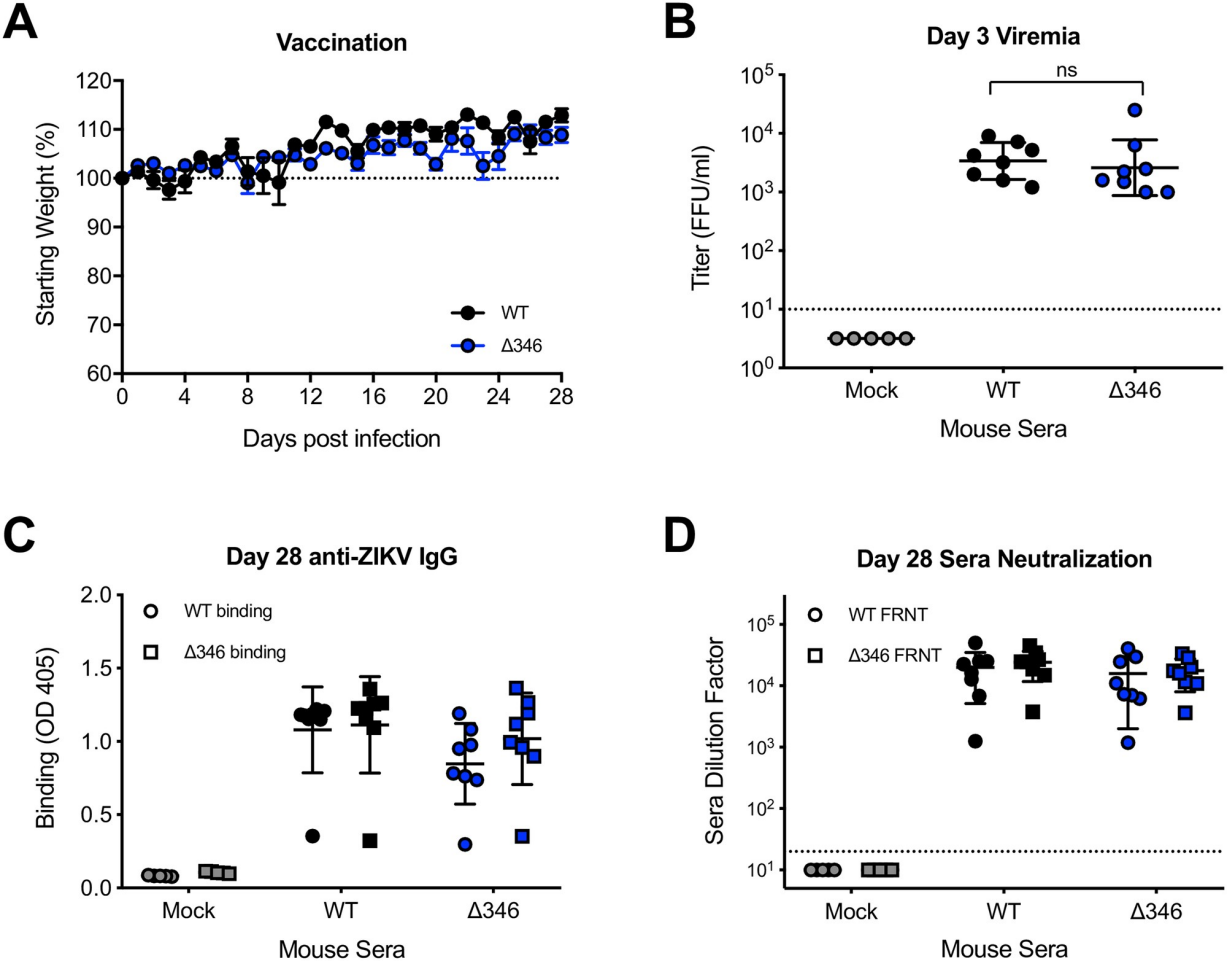
Δ346 mutant induces antibody response similar to WT ZIKV infection. C57BL/6J mice were pretreated with anti-IFNAR1 blocking antibody, infected with 10^3^ FFU WT or Δ346 ZIKV via footpad infection, and (A) monitored for daily weight loss (n = 8 for each group). (B) On day three post infection, blood was collected and titered to measure serum viremia (n = 5 mock infected mice, n = 8 for both infected groups). (C-D) Day 28 immune sera was collected and analyzed for (C) IgG antibody binding to WT and Δ346 ZIKV via ELISA and (D) neutralization titer analysis via C6/36-based focus reduction neutralization test (FRNT). Y-axis represents serum dilution factor required to neutralize 50% of infectious virus. One-factor ANOVA followed by Tukey’s multiple comparisons test was conducted on log-transformed titer data. Two-factor ANOVA followed by Tukey’s multiple comparisons test was conducted on binding and neutralization data. * represents *p* < 0.05. Dotted line represents 100% starting weight or limit of detection. Data points not detected are plotted at half the limit of detection. Error bars represent (A) standard error of the mean or (B-D) standard deviation.

As the Δ346 mutant is attenuated in neuropathogenesis, mutations that shorten the ZIKV CD-loop may be incorporated into future live-attenuated vaccines. We have previously shown that while the Δ346 virus is structurally destabilized, viruses produced in cell culture are morphologically similar to WT ZIKV [24]. To determine if Δ346 ZIKV infection elicits an antibody response similar to that of WT ZIKV infection, we infected C57BL/6J mice pretreated with an IFNAR-blocking antibody and analyzed the anti-ZIKV antibodies. Mice infected with WT or Δ346 ZIKV did not show any signs of disease, but supported acute viral replication with similar serum viremia on day three post infection (p>0.05) (Fig 6A and 6B). We next evaluated the ability of WT and Δ346 ZIKV immune sera to bind and neutralize both WT and Δ346 ZIKV viruses. WT ZIKV immune sera had similar levels of ZIKV-reactive IgG compared to Δ346 sera, and both sera bound both viruses similarly (p>0.05) (Fig 6C). Despite lower levels of total ZIKV antibodies, sera from Δ346-infected mice efficiently neutralized both viruses, with neutralization titers similar to those of WT ZIKV sera (p>0.05) (Fig 6D). Importantly, these data show that the Δ346 mutant maintains antigenicity and elicits neutralizing antibodies against WT ZIKV suggesting potential application as a part of a live-attenuated vaccine approach.

## Discussion

Having previously characterized the extended ZIKV CD-loop as an important determinant of stability and virulence [24], here we further examined the shortened CD-loop mutant virus. The Δ346 mutant displays a mild growth defect in a neuronal cell line suggesting capacity to replicate in the brain. In addition, we confirmed *in vivo* attenuation via the peripheral route of infection in terms of dissemination to the CNS as well as replication in the brain relative to WT ZIKV. The Δ346 mutation is genetically stable after *in vivo* infection. However, Δ346 virus sequenced from the brains of mice reveal secondary site mutations near the CD-loop whose functions are unknown, but may compensate for viral stability or neuropathogenicity. The secondary site mutations identified in this study may inform future structure-function studies of the CD-loop in regards to ZIKV virion stability and pathogenesis, identifying key CD-loop contacts critical in the ZIKV virion.

After intracranial infection, the Δ346 mutant was highly attenuated, demonstrating that in addition to defects in neuroinvasion, the CD-loop mutant causes delayed encephalitic disease when delivered directly to the CNS. While the CD-loop mutant displays attenuation, it is capable of eliciting antibodies that neutralize WT ZIKV, suggesting it still maintains proper ZIKV antigenicity. While we do not know the mechanism of the Δ346 mutant attenuation beyond virion stability, the shortened CD-loop may alter cellular tropism, particularly in the brain, resulting in fewer infected cells and delayed disease. Defining the cell types infected by the Δ346 mutant and WT ZIKV in tissues, specifically the brain, may provide insights into the decreased disease phenotype.

The viral determinants that allow ZIKV and other neurotropic flaviviruses to invade and replicate in the CNS are not fully understood [6, 7, 13]. Here, we show that shortening of the CD-loop in the ZIKV envelope results in delayed neuroinvasion in immunocompromised mice. This delayed neuroinvasion may be due stochastic differences in intra-host evolution of the Δ346 virus developing secondary site mutations that increase fitness in the CNS.

Neurotropic flaviviruses WNV, JEV, and TBEV also share an extended CD-loop within the envelope protein relative to hemorrhagic flaviviruses such as DENV [23]. Shortening the CD-loop in other flaviviruses would be important in understanding if the CD-loop modulates stability and neurovirulence in other viruses. Within ZIKV strains, there is variation in the pathogenicity of ZIKV isolates. Multiple groups have shown that Asian ZIKV strains, such as the H/PF/2013 strain used in this study, are less neurovirulent than African strains [30, 31]. The extended CD-loop is conserved among all ZIKV strains, thus we expect that shortening the CD-loop in African ZIKV strains will also attenuate the virus, though the effects may be more modest due to other determinants driving their increased neuropathogenicity.

Targeting the CD-loop may be a broadly applicable approach for flavivirus attenuation. Groups have shown that glycosylation of the WNV envelope protein has been well studied for its role in neurotropism, however, it is possible the extended CD-loop is an additional viral determinant of neurotropism [16, 17]. Although speculative, a WNV CD-loop mutant could be an important tool for studying CD-loop-mediated neurotropism, as WNV causes encephalitic disease in immunocompetent mice [16, 17]. Relative to DENV, both ZIKV and WNV have an extended CD-loop; however, the identity of residue 346 varies, with alanine in ZIKV and valine in WNV. It remains unclear whether the length of the CD-loop or the identity of the residue at position 346 is critical for pathogenesis. While several flavivirus vaccines exist, the YFV vaccine is occasionally associated with rare complications [32–35]. Though YFV is non-neurotropic, it contains an extended CD-loop [23] and incorporation of the Δ346 mutation into the YFV vaccine may further increase safety, while preserving antigenicity and efficacy. Live-attenuated vaccines resulting from deletions, such as a shortened CD-loop, are also more likely to be genetically stable than attenuating substitution mutations.

There are limitations of studying ZIKV pathogenesis in current animal models, including the necessity of using immunocompromised mice due to the strong restriction of ZIKV by murine interferon signaling [29, 36]. Circumventing the interferon restriction, via genetic knockout mice or antibody blockade of the interferon response, permits ZIKV replication, but limits our ability to study the intact immune response to infection. Additionally, the use of immunocompromised mice with delayed viral clearance is anticipated to increase the likelihood of accumulating secondary site mutations which may contribute to restored replication fitness and pathogenesis. Mouse adapted ZIKV viruses and use of humanized or human *STAT2* knock-in mice may serve as better future models to understand innate and adaptive responses [37]. As these models become more available, it will be important to study the kinetics, pathogenesis, and genetic stability of Δ346 ZIKV in these more relevant, immunocompetent models. These models will also be important in testing the genetic stability of Δ346 ZIKV in an immunocompetent model.

Overall, this work highlights the importance of the extended CD-loop for ZIKV neuropathobiology. The extended CD-loop is an important determinant for ZIKV dissemination and CNS invasion, but also crucial for efficient replication in the brain. However, shortening of the CD-loop does not abolish neuropathogenicity, highlighting the multifaceted nature of ZIKV neurovirulence. Additionally, shortening of the ZIKV CD-loop, while attenuating the virus, maintains antigenicity and may be utilized in next-generation vaccine design in combination with additional attenuating mutations in the genome that reduce chance of disease.

## Methods

### Viruses and cells

Viruses were generated using a ZIKV H/PF/2013 infectious clone as described previously [24, 38]. Low passage virus stocks were produced in C6/36 cells. C6/36 cells (ATCC CRL-1660) were maintained in Minimum Essential Medium, supplemented with 5% fetal bovine serum (FBS), 1% nonessential amino acids (NEAA), and 1% antibiotic-antimycotic (AA) and grown in 5% CO_2_ at 32°C. SH-SY-5Y cells (ATCC CRL-2266) were maintained in MEM:F12 supplemented with 10% FBS and 1% AA and grown in 5% CO_2_ at 37°C.

### Virus titering and immunostaining

Samples were titered on C6/36 cells as the particle to FFU ratio is more similar between WT and Δ346 ZIKV than when titered on Vero cells [24]. Viruses were serially diluted and added to C6/36 monolayers for one hour. Cells were then overlaid with OptiMEM supplemented with 1% methylcellulose, 2% FBS, 1% NEAA, and 1% AA and incubated at 32°C for 4–5 days. Cells were gently washed with PBS and fixed in cold 50% acetone + 50% methanol, then immunostained with either mouse anti-E MAb 4G2 or human anti-E MAb 1M7, HRP-labeled secondary antibody, and developed with KPL TrueBlue substrate.

### SH-SY5Y growth curve

Plates were seeded with SH-SY5Y cells one day prior to infection. Cells were infected in triplicate at an MOI of 1 for two hours, then washed with PBS to remove unbound virus, and replaced with fresh media. Every 24 hours, supernatant was sampled, and immediately frozen at -80°C. Samples were titered on C6/36 cells as described above. Two-factor ANOVA followed by Sidak’s multiple comparison was conducted on log-transformed data. Experiment was performed once.

### Ethics statement

This study was carried out in accordance with the recommendations for care and use of animals by the Office of Laboratory Animal Welfare (OLAW), National Institutes of Health. All animal work was performed in strict adherence to the Institutional Animal Care and Use Committee (IACUC) and University of North Carolina at Chapel Hill policy. The protocol [#16–090] was approved by the UNC’s IACUC (Permit Number A-3410-01).

### Mouse infections

Footpad infections were performed in 8- to 10-week-old male and female IFNAGR^-/-^ mice on C57BL/6 background with 10^3^ or 10^4^ FFU of WT or Δ346 ZIKV in 10ul diluted in PBS into the left hind footpad (n = 13 for each group). Infected mice were monitored daily for weight loss and clinical signs of disease. On day six post infection, a subset of mice were euthanized and perfused with PBS for tissue analysis. The remainder of mice were euthanized via isoflurane overdose and cervical dislocation upon losing 20% of their starting body weight or showing signs of severe disease and tissues were collected for titer analysis. No mice died prior to humane euthanasia. Tissues were homogenized in 1ml PBS, centrifuged to pellet debris, immediately frozen at -80°C, then clarified supernatant was used for virus titering as described above. Survival curves were analyzed by log-rank test. Log-transformed titer data was analyzed by Mann-Whitney test.

Intracranial infections were performed in 5- to 7-week-old male and female C57BL/6J or IFNAGR^-/-^ mice with 10^3^ FFU of WT or Δ346 ZIKV in 20ul diluted in PBS into the left cerebral hemisphere via insulin syringe while under brief isoflurane anesthesia and monitored as described above (For IFNAGR^-/-^ mice, n = 11 and 14 respectively. For C57BL/6J mice, n = 15 and 14, respectively). Mice were euthanized on days two, six, or upon losing 20% of their starting weight, and tissue was collected and titered as described above. No mice died prior to humane euthanasia. Survival curves were analyzed by log-rank test. Log-transformed titer data was analyzed by two-factor ANOVA followed by Sidak’s multiple comparisons.

Antibody response experiments were performed in five-week-old female C57BL/6J mice, which were given 1mg of anti-IFNAR1 antibody (MAR1-5A3, Bio X Cell) via intraperitoneal injection one day prior to footpad infection and monitored as described above (n = 5 mock infected and n = 8 for each ZIKV infected group). Blood was collected on day three post infection via submandibular bleed, allowed to clot for at least 10 minutes and clarified by centrifugation prior to storage at -80°C. Mice were euthanized on day 28 post infection for terminal blood collection. Log-transformed titer data was analyzed by one-factor ANOVA followed by Tukey’s multiple comparisons. All mouse experiments were performed once.

### Sequencing ZIKV envelope

Virus stock RNA was isolated via Viral RNA Mini Kit (Qiagen). Total brain RNA was isolated from brain homogenates and TRIzol LS (Invitrogen) and Direct-zol RNA MiniPrep kit (Zymo Research). cDNA was reverse transcribed using SuperScript III (Invitrogen) using random primers. The ZIKV envelope sequence was PCR amplified, and PCR amplicon was sequenced via Sanger sequencing and analyzed in Geneious (Version 11.0.04).

### Enzyme-Linked Immunosorbent Assay (ELISA)

96-well high bind plates were coated with human MAb EDE1 C10, previously shown to bind WT and Δ346 ZIKV equally [24]. Plates were blocked with 3% non-fat milk, then viral antigen (WT or Δ346 ZIKV) was captured for one hour at 37°C. Mouse immune sera was diluted 1:1000 then allowed to bind to captured virus for one hour at 37°C. Anti-mouse-IgG alkaline phosphatase labeled secondary antibody was added, plates were developed using p-nitrophenyl phosphate, and color changes were quantified via spectrophotometry. ELISA data was analyzed by two-factor ANOVA followed by Tukey’s multiple comparisons.

### Focus reduction neutralization test (FRNT)

Plates were seeded with C6/36 cells one day prior to neutralization assay. Mouse immune sera were serially diluted four-fold beginning at 1:20, then mixed with WT or Δ346 ZIKV viruses diluted to ~45 FFU/well. Virus:Ab mixture was incubated for one hour at 32°C, added to C6/36 cells and incubated for an additional hour at 32°C. Overlay was added and cells were incubated for 4–6 days, then fixed and immunostained as described above. FRNT data was analyzed by two-factor ANOVA followed by Tukey’s multiple comparisons.

## Supporting information

S1 Data Raw data of included figures. Microsoft Excel file containing data used to generate figures in Prism (version 8.0.1). Individual sheets correspond to each figure panel. ZIKV titer data provided as the log transform of foci forming units per ml or gram of tissue (FFU/ml or FFU/g). Limits of detection are also provided. Mouse weight data provided in grams and as percent of starting weight. Includes full ZIKV envelope protein amino acid sequence for each sample.
(XLSX)
